# Transcriptomic responses to high water temperature in two species of Pacific salmon

**DOI:** 10.1111/eva.12119

**Published:** 2013-11-12

**Authors:** Ken M Jeffries, Scott G Hinch, Thomas Sierocinski, Paul Pavlidis, Kristi M Miller

**Affiliations:** 1 Centre for Applied Conservation Research Department of Forest and Conservation Sciences, University of British ColumbiaVancouver, BC, Canada; 2 Centre for High-Throughput Biology Department of Psychiatry, University of British ColumbiaVancouver, BC, Canada; 3 Fisheries and Oceans Canada, Molecular Genetics SectionNanaimo, BC, Canada

**Keywords:** climate change, ecological genomics, *Oncorhynchus gorbuscha*, *Oncorhynchus nerka*, premature mortality, spawning migration

## Abstract

Characterizing the cellular stress response (CSR) of species at ecologically relevant temperatures is useful for determining whether populations and species can successfully respond to current climatic extremes and future warming. In this study, populations of wild-caught adult pink (*Oncorhynchus gorbuscha*) and sockeye (*Oncorhynchus nerka*) salmon from the Fraser River, British Columbia, Canada, were experimentally treated to ecologically relevant ‘cool’ or ‘warm’ water temperatures to uncover common transcriptomic responses to elevated water temperature in non-lethally sampled gill tissue. We detected the differential expression of 49 microarray features (29 unique annotated genes and one gene with unknown function) associated with protein folding, protein synthesis, metabolism, oxidative stress and ion transport that were common between populations and species of Pacific salmon held at 19°C compared with fish held at a cooler temperature (13 or 14°C). There was higher mortality in fish held at 19°C, which suggests a possible relationship between a temperature-induced CSR and mortality in these species. Our results suggest that frequently encountered water temperatures ≥19°C, which are capable of inducing a common CSR across species and populations, may increase risk of upstream spawning migration failure for pink and sockeye salmon.

## Introduction

Many fish populations are chronically or acutely exposed to water temperatures outside their species-or population-specific preferred temperature ranges and these occurrences are expected to become more frequent due to climate change. Water temperature has a profound influence on fish physiology, which includes temperature-dependent changes in metabolic rates (Brett 1971). Aerobic scope, the difference between routine and maximum oxygen consumption during aerobic activity (Fry 1947), has been recommended as a tool to determine the thermosensitivity of fish species (Portner 2002; Portner and Knust 2007; Farrell et al. 2008). The aerobic scope concept suggests that there is an optimal temperature for aerobic performance, and temperatures exceeding the optimal temperature will result in reduced aerobic performance and an eventual collapse in aerobic capabilities [at their ‘critical temperature’, (Portner 2002)]. As water temperature increases towards the critical temperature for aerobic scope, cellular functions are maintained and protected by heat shock proteins and antioxidant defences (Portner 2002; Kassahn et al. 2009). This suggests that a cellular stress response (CSR), which would involve differential regulation of genes associated with protein folding and antioxidant defences, would be activated when a fish is experiencing a thermal environment approaching its critical temperature for aerobic scope. Often changes in the expression of genes involved in the CSR can begin to be detected at temperatures below where effects at the whole organism level can be observed. As environmental exposures to temperature extremes are often chronic in duration, the length of time that a fish can maintain its CSR is an important factor in determining how a species will respond to high water temperatures and may depend on the thermosensitivity of the fish species or population. For example, eurythermal species, such as the annual killifish (*Austrofundulus limnaeus*) and longjaw mudsucker (*Gillichthys mirabilis*) have been shown to maintain a CSR for periods of weeks (Podrabsky and Somero 2004; Logan and Somero 2010), which may not be possible in other less thermally tolerant species. Characterizing the CSR of a species at ecologically relevant temperature exposures is useful for attempting to understand the ability of that species to successfully respond to future warming and current climatic extremes.

Ecologically and economically important Pacific salmon species (*Oncorhynchus* spp.) are of concern because they experience some of the warmest water temperatures during their upstream spawning migration – a crucial life-history stage. Migration during warm water periods is associated with increased incidences of premature mortality (*en route* and pre-spawn mortalities; Gilhousen 1990; Heard 1991; Keefer et al. 2008; Taylor 2008; Keefer et al. 2010; Macdonald et al. 2010). Pacific salmon populations at the southern periphery of their species’ distributions are particularly at risk of population declines or extirpation due to warming water temperatures (Beamish et al. 1997). As many southern populations already frequently experience temperatures that result in premature mortalities (e.g. Keefer et al. 2008; Martins et al. 2011), warming river temperatures will likely contribute to declines in many Pacific salmon populations in British Columbia (B.C.), Canada, and Washington, Oregon and California, USA.

The Fraser River, B.C., with a drainage basin of approximately 240 000 km^2^, is Canada's largest and most economically important salmon-producing river and is near the southern extent of the distributions of pink (*Oncorhynchus gorbuscha*) and sockeye (*Oncorhynchus nerka*) salmon. Peak and average Fraser River summer water temperatures have been increasing over recent decades, with 13 of the past 20 summers being the warmest on record (Patterson et al. 2007; data available at http://www.pac.dfo-mpo.gc.ca/index-eng.html). This has resulted in many Fraser River Pacific salmon populations performing their upstream spawning migration in relatively warm conditions, which has led to high *en route* mortality (Hinch et al. 2012). Management agencies often use a threshold temperature of 18°C (Macdonald et al. 2000) for determining when spawning migrations are considered more difficult due to temperature stress for all Fraser River Pacific salmon populations; a temperature that has been reached or exceeded in 8 of 9 years from 2005 to 2013. Temperatures above 18°C have been shown to be capable of inducing a CSR in some sockeye salmon populations (Jeffries et al. 2012a); however, it is still unknown whether common temperature-induced CSRs exist among Pacific salmon species and whether prolonged activation of a CSR is detrimental to adult Pacific salmon. Characterizing a common temperature-induced CSR across species and populations will aid in pink and sockeye salmon management through the development of molecular biomarkers of temperature stress that can be used as monitoring tools to assess conditions that are physiologically stressful for various populations of migrating Pacific salmon.

Pacific salmon are semelparous and die after spawning; consequently, they are senescing as they mature during their upstream migration, which complicates interpreting the physiological effects of elevated water temperature on Pacific salmon. It is currently unknown whether senescence, and consequently proximity to final maturation, influences the temperature-induced CSR of adult Pacific salmon. However, because expression of genes involved in metabolism and protein biosynthesis have been shown to be affected by both temperature and maturation in wild Pacific salmon (Miller et al. 2009; Jeffries et al. 2012a), advanced senescence and reproductive status could alter the ‘normal’ responses of these processes in thermally stressed fish.

In this study, different populations and species of wild-caught adult pink and sockeye salmon from the Fraser River were experimentally treated to an ecologically relevant ‘cool’ (13 or 14°C depending on the population studied) or ‘warm’ (19–1°C above the threshold temperature used by management agencies) water temperature to uncover common transcriptomic responses to elevated water temperature. We used pink and sockeye salmon as our model as these well-studied species suffer increased *en route* mortality during migration in warm water conditions and will likely be adversely affected by future increases in water temperature. Because it is known that 19°C is physiologically stressful for sockeye and pink salmon, we hypothesized that chronic exposure to 19°C would result in higher incidences of premature mortality and would elicit a CSR detectable in the transcriptomes of these species. We predicted that a CSR signature resulting from exposure to a 19°C temperature treatment would include an up-regulation of heat shock proteins and genes involved in an oxidative stress response along with the differential regulation of genes involved in protein biosynthesis, consistent with previous work (Jeffries et al. 2012a), and predicted that this response would be common across two species and different populations of Pacific salmon. Additionally, we predicted that senescence and maturation would influence the temperature-induced CSR by affecting metabolism and protein biosynthesis when compared with fish that were less mature. The goal of this study was to characterize a common temperature-induced CSR that can be used to develop molecular tools to identify periods of temperature stress across populations and species of pink and sockeye salmon.

## Materials and methods

### Fish collection and holding conditions

Adult Pacific salmon were collected by beach seine from the mainstem of the Fraser River, B.C., 5–7 September 2007 (*n* = 130; mixed populations targeting summer-run sockeye salmon), and the major Fraser River tributary Harrison River, B.C., 15–18 September 2008 (*n* = 128; late-run sockeye salmon) and 22–24 September 2009 (*n* = 156; Lower Fraser River pink salmon), ~10 km upstream of the confluence of the Fraser and Harrison Rivers. Both collection sites were ~110–120 km upstream of the mouth of the Fraser River. Each year, fish were transported by truck in aerated tanks to the Fisheries and Oceans Canada Cultus Lake Laboratory, near Chilliwack, B.C., where they were randomly distributed at equal densities and sex ratios among four to eight 8000 L aerated tanks with 10–12°C sand-filtered and UV-sterilized water. Sexes were determined visually upon arrival at the Cultus Lake Laboratory and confirmed during autopsies conducted at the end of the holding period. Each tank contained a submersible pump that created a water flow of approximately 0.3 m s^−1^ into which the fish were able to maintain position by continuous, gentle swimming. Because there are ~150 genetically distinct populations of sockeye salmon that migrate to different natal streams throughout the Fraser River watershed, fish in 2007 and 2008 were passive integrated transponder-tagged and an adipose fin clip was taken for DNA stock identification (see below).

Fish were given 2–6 days at 10–12°C to recover from transport, after which they appeared vigorous and without external signs of disease. Water temperatures were subsequently raised at a rate of 2–3°C day^−1^ until the test temperatures of 13 or 14°C (cool treatment) and 19°C (warm treatment) were reached. After 5–7 days at the test temperature, a period of time that many populations are exposed to warm water temperatures while migrating through the Lower Fraser and Fraser Canyon regions of the Fraser River (English et al. 2005), small pieces of gill tissue were non-lethally sampled from surviving fish in both temperature groups to determine the effect of water temperature on gene expression. Gill tissue samples were only taken from fish that were vigorous and maintaining position in the current and were immediately flash frozen in liquid nitrogen and stored at −80°C until analysis. Fish that were observed to become moribund were immediately removed and enumerated throughout the holding studies. Survival patterns were continuously monitored post-sampling until the termination of the experiments. In 2008 and 2009, rapid mortality of females in the warm temperature groups required a reduction in temperatures to ~7–9°C post-sampling in a subset of tanks to improve survival. It is important to note that this would not affect the gene expression profiles as the gill samples were taken while the fish were still exposed to the warm water temperature.

DNA identification (Beacham et al. 2005) confirmed that the sockeye salmon used in the microarray study were summer-run populations in 2007 and were a late-run population in 2008. Because the summer-run fish used in the present study have similar thermal tolerances in terms of aerobic scope, and face similar migration challenges in terms of historical temperature, distance and elevation gain (Eliason et al. 2011), we pooled the summer-run stocks of Chilko, Horsefly and Mitchell. Fraser River pink salmon are generally divided into Lower and Upper Fraser River populations, which can be distinguished based on migration timing and capture location (Heard 1991; Crossin et al. 2003), but not by DNA identification (T.D. Beacham, personal communication). The pink salmon that spawn in the Harrison River (used in the present study) belong to the Lower Fraser River population complex. Specific details of the fish populations studied and the temperature reatment experiments for each year are provided in Table 1.

**Table 1:** Population and experimental design details for the sockeye and pink salmon held at a warm or cool temperature in three different temperature holding experiments conducted from 2007 to 2009.

Species	Year	Run timing	Population*	Treatment duration	Temperature (°C)	Sex	*n*	Date of Sampling
Sockeye Salmon	2007	Summer-run	Chilko/Horsefly/Mitchell† (~650–800 km)	7 days	19	M	10	21 September 2007
						F	3	
					14	M	3	
						F	5	
Sockeye Salmon	2008	Late-run	Harrison‡ (~115 km)	5 days	19	M	11	1 October 2008
						F	3	
					13	M	11	
						F	8	
Pink Salmon	2009	N/A	Lower Fraser River§ (~105–120 km)	5 days	19	M	11	5 October 2009
						F	11	
					13	M	11	
						F	11	

* Freshwater distances travelled by those populations to reach natal spawning grounds during spawning migrations in parentheses.

† 2007 Peak spawning – Chilko: September 28–October 3, Horsefly: September 5–15, Mitchell: Not available.

‡ 2008 Peak spawning – November 11–13.

§ 2009 Peak spawning – Weaver Creek spawning channel: October 13–16.

### Microarray analysis

Total RNA was purified from individual fish gill tissue using Magmax™-96 for Microarrays Kits (Ambion Inc., Austin, TX, USA) with a Biomek FXP (Beckman-Coulter, Mississauga, ON, Canada) automated liquid-handling instrument. Gill tissue (≤0.5 mg) from each fish was homogenized with stainless steel beads in TRI-reagent (Ambion Inc.) on a MM301 mixer mill (Retsch Inc., Newtown, PA, USA). 1-bromo-3-chloropropane was added to the homogenized sample and aliquots of 100 μL of the aqueous phase were pipetted into 96-well plates. Extractions were carried out using the ‘Spin Procedure’ following manufacturer's instructions. In the final step, RNA was eluted and yield was determined by measuring the A260 using a DTX 880 Spectrophotometer (Beckman-Coulter). The RNA purity was assessed by measuring the A260/A280. Solutions of RNA were stored at −80°C until amplification.

Eleven microlitres of total RNA was purified, amplified and amino allyl-modified using Amino Allyl MessageAmp™ II-96 kits (Ambion Inc.) following manufacturer's instructions. Amplification of the RNA was necessary as non-lethal sampling of gill tissue prevents the collection of large amounts of gill. Because of variable concentrations of extracted total RNA due to different sizes of gill tissue available for the extractions, we used the maximum 11 μL of total RNA for the amplification procedure and normalized the aaRNA concentrations for the subsequent procedures. The aaRNA samples were then purified, lyophilized and resuspended in 12 μL of DNAse/RNAse-free water prior to the dye coupling reactions. Samples were labelled with Alexa dyes (Invitrogen, Carlsbad, CA, USA) in the dark. After 1 h incubations at room temperature, the reactions were quenched using 4 m hydroxylamine and the samples were purified, eluted in 35 μL of aaRNA elution buffer and quantified using a NanoDrop Spectrophotometer ND-1000 (Nanodrop Products, Wilmington, DE, USA). Treatment samples were labelled with Alexa 555 and reference samples, comprised of a pool of RNA from all the fish used in the study, were labelled with Alexa 647. Labelled samples were stored at −80°C until used for hybridization.

A total of 98 arrays were run using the cGRASP 4x44K Salmonid Oligo Arrays (Agilent Technologies, Santa Clara, CA, USA; http://web.uvic.ca/grasp/microarray/array.html). Each sample was run on a single array against a reference control; to minimize technical artefacts, labelling reactions were performed simultaneously and individuals were randomized between slides and hybridization days. Prior to hybridization, dye labelled treatment and reference samples (825 ng each) were combined along with 1.2 μL 25× fragmentation buffer, 6 μL blocking agent and nuclease-free water to bring the final volume to 30 μL. The fragmentation mix was incubated at 60°C for 30 min and was stopped by adding 30 μL of 2× GEx hybridization buffer HI-RPM. Samples were briefly centrifuged, placed on ice and 55 μL of the mix was loaded into hybridization chambers in a Tecan-HS4800 Pro Hybridization Station (Tecan Trading AG, Männedorf, Switzerland). The slides were washed with Prehybridization Buffer (Agilent Technologies) at 65°C prior to sample loading. Samples were hybridized to the arrays for 17 h at 65°C, followed by a wash with Gene Expression Wash Buffer 1 (Agilent Technologies) at room temperature, a wash with Gene Expression Wash Buffer 2 (Agilent Technologies) at 37°C and 2 min drying at 30°C. All steps from washing, hybridization and slide drying were carried out automatically. Fluorescent images were scanned using a Tecan LS Reloaded Scanner (Tecan Trading AG) and the analysis software Array-Pro Analyser (Media Cybernetics, Inc., Bethesda, MD, USA). The images were quantified using Imagene (BioDiscovery, El Segundo, CA, USA). Each slide was normalized in BASE using the LOESS method. A detailed description of how slide quality and outliers were assessed can be found in supplemental material from Miller et al. (2011). The microarray data have been deposited in the Gene Expression Omnibus (http://www.ncbi.nlm.nih.gov/geo/) with the accession number GSE42558.

### Data analysis

Log-Rank tests with the Holm-Sidak method for *post hoc* pairwise comparisons were used to statistically compare cumulative mortality curves for both sexes and temperature treatments in each year. Fish that survived the temperature treatment periods were accounted for by censoring. Principal component analysis (PCA) was conducted to identify the major transcriptional trajectories in the data. Missing values were imputed for the PCA using the mean expression of that gene for the experiment. Genes that had more than 50% missing values were filtered out. The PCA method provides a ranking of genes and individuals characterizing their contribution (in terms of variance) to each principal component (PC; i.e. the loading). Both positive and negative loadings were considered. The relationships between an individual's position on a PC axis and the temperature treatments, sex and species were assessed using Mann–Whitney *U* (MWU) tests. The relationships between an individual's position on a PC axis and sampling year and populations (in 2007) were assessed using Kruskal–Wallis (KW) tests, which is an extension of the MWU test to examine more than two experimental groups. Pearson's correlations were used to examine the relationship between an individual's position on a PC axis and how long each fish survived post-sampling.

Because there were subtle differences in experimental designs each year and there can be considerable variation in the condition of returning salmon between years and populations, direct comparisons between experiments conducted in the different years were not considered appropriate when performing the supervised analyses. Additionally, there is the potential for species differences in the hybridization to the array, which may affect the interpretation of differences in gene expression profiles between the different salmon species. Therefore, we conducted two-factor anovas for each year with temperature and sex as factors, and used a Venn diagram approach (Kammenga et al. 2007) to infer common transcriptomic changes for each run-time group and species. This approach is useful for determining commonalities between multiple datasets and has been used previously in salmonid microarray studies (Quinn et al. 2011a,b2011b). Genes were considered significantly different between treatments at a conservative *q* < 0.01 [the false discovery rate (FDR) analogue of the *P*-value]. It is important to note that multiple copies of the same gene may reflect either different regions of the same gene or duplicated copies of that gene due to pseudo-tetraploidy in the salmon genome (Quinn et al. 2011a); hence, we included all copies of each gene that was significant for our analyses; however the conservative *q*-value threshold reduced some of that redundancy in the gene lists. Pearson's correlations were used to examine the relationship between gene expression levels that were common among experiments and post-sampling survival within the 19°C treatment for each year. Statistical analyses were performed using R (R Development Core Team 2008), SigmaPlot (SYSTAT Software, Inc., Chicago, IL, USA) or MultiExperiment Viewer (MeV; Saeed et al. 2006).

Functional analysis of gene expression profiles was performed using the receiver operator characteristic (ROC) scoring method in ErmineJ (Lee et al. 2005) on the absolute values of the gene rankings from each PCA. The ROC method is a non-threshold method performed on all gene rankings, which may be more robust than using raw gene scores. All three categories of the GO hierarchy (Biological Process, Molecular Function and Cellular Component) were considered, limited to groups with 5–100 genes. However, only the categories Biological Process and Molecular Function are presented. The ‘best-scoring replicate’ method was used in ErmineJ to handle repeated measurements of the same gene. Gene sets in the ErmineJ analyses were considered significant at Benjamini-Hochberg FDR < 0.05. Functional redundancies in the GO term lists were reduced using Revigo (Supek et al. 2011).

## Results

### Mortality patterns

**Figure 1:**
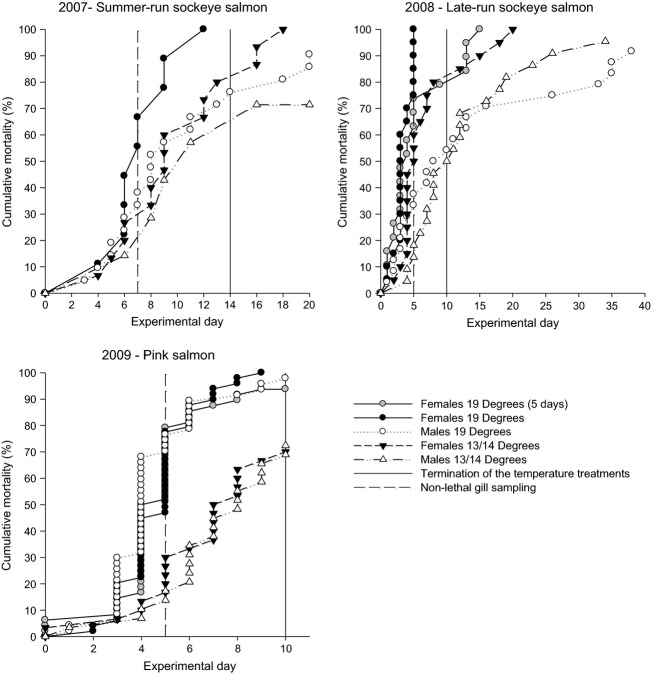
Observed cumulative mortality of male and female pink and sockeye salmon held at a warm or cool temperature for each experimental year. The dashed line indicates when the fish were sampled for gill tissue and the solid line indicates when the temperature treatments were terminated. In 2007 and 2008, water temperatures were reduced to ~7–9°C after the termination of the temperature treatments. Due to rapid mortality of females in 2008 and 2009, some of the warm temperature treatment tanks were lowered to ~7–9°C after 5 days to reduce mortality. Mortality patterns of females from these tanks are indicated in grey.

Overall, there was substantial mortality (i.e. ≥60%) after 10 days at 19°C (Fig. 1). There was higher overall mortality at 19°C for both sexes of pink salmon in 2009 (Log-Rank test; *P *<* *0.001) and for sockeye salmon females in 2008 (Log-Rank test; *P *<* *0.001) compared with fish held at 13°C. Female sockeye salmon in 2007 had higher mortality at 19°C than females at 14°C (Log-Rank test; *P *<* *0.03); however, this difference was not significant after multiple test correction. There was sex-specific mortality in 2008 with female sockeye salmon having higher overall mortality than males. This sex-specific mortality pattern was also observed in 2007; however, the pattern was not significant after multiple test correction. Sex-specific mortality patterns were not detected in pink salmon. Female mortality tended to decrease after the temperature treatments were dropped to cooler temperatures (~7–9°C).

### PCA among years and populations

**Figure 2:**
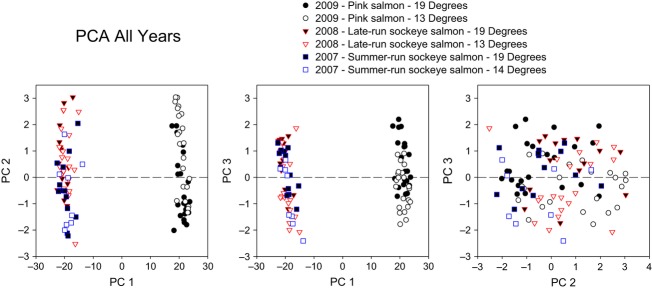
Position of each individual fish (*n* = 98) along the first three principal component (PC) axes for the principal component analysis (PCA) conducted on all fish from each experimental year (PC1 explained 35.0% of the variance, with PC2 and PC3 explaining 6.0% and 4.9% of the variance respectively).

Based on the PCA performed on all fish and all years, PC1 explained 35.0% of the variance, with 6.0% and 4.9% of the variance explained by PC2 and PC3 respectively. The experiment year was strongly associated with PC1 (KW, *P* << 1.0E^−20^), with distinct separation between the pink salmon sampled in 2009 and the sockeye salmon sampled in 2007 and 2008 (Fig. 2). Species was also significantly associated with PC1 (MWU, *P *<* *1.0E^−15^) further indicating that the strongest pattern in the PCA was the differences between pink and sockeye salmon. Functional analysis indicated that processes involved in oxidative stress, protein catabolism, lipid metabolism and calcium ion binding were significantly enriched (FDR < 0.05) in the PC1 gene list (Table S1).

Functional analysis suggests that PC2 may have been associated with cell survival as processes involved in apoptosis and overall protein breakdown were significantly enriched (FDR < 0.05) in the PC2 gene list. These processes were up-regulated in fish on the negative end of the PC axis.

While the temperature treatments were significantly associated with PC1 (KW, *P *=* *0.03) and PC2 (KW, *P *=* *0.002), they were most strongly associated with PC3 (KW, *P *=* *0.00016) with the fish held at 19°C generally being located on the positive end of the PC3 axis (Fig. 2). Functional analysis indicated that processes involved in the cell cycle and proliferation, cell signalling pathways and immune response were significantly enriched (FDR < 0.05) in the PC3 gene list (Table S1). Sex was not significantly associated with any of the PC axes (MWU, *P *>* *0.05).

### PCA on individual years

When separate PCAs were performed on data from each year, similarities among the different year's experiments emerged. Each year, the temperature treatments were significantly associated with PC1 (Table 2). However, the ranking of individuals along the PC1 axes were significantly correlated with the length of time salmon survived post-sampling (Figure S1A; 2007: *r *=* *−0.67, *P *<* *0.001; 2008: *r *=* *−0.71, *P *<* *0.00001; 2009: *r *=* *0.50, *P *<* *0.001). Therefore, PC1 (explaining 12.3–13.9% of the variance in the data) is likely associated with mortality. Indeed, functional groups involved in cell proliferation and apoptosis were enriched in the PC1 gene lists for each year (Table S2), similar to PC2 in the PCA that included all 3 years. Additionally, biological processes involved in metabolism, protein catabolism, ion homeostasis, G-protein coupled receptor signalling, oxidative stress, antigen binding and immune response were also enriched in the PC1 gene list for each year.

**Table 2:** *P*-values from Mann–Whitney *U* or Kruskal–Wallis tests to determine the relationship between temperature, sex and stock (if appropriate) and the first four principal components, along with the variance explained, from the principal component analysis conducted separately on each experimental year.

Species	Year	Run timing	Population	Principal component	Variance explained (%)	Temperature	Sex	Stock
Sockeye Salmon	2007	Summer-run	Chilko/Horsefly/Mitchell	1	12.44	**2.46E-02**	7.56E-02	2.85E-01
				2	10.74	4.14E-01	**5.96E-03**	**3.13E-03**
				3	10.40	8.60E-01	8.60E-01	2.34E-01
				4	6.88	**1.97E-05**	6.45E-01	2.36E-01
Sockeye Salmon	2008	Late-run	Harrison Rapids	1	12.25	**2.88E-02**	1.54E-01	
				2	8.16	4.83E-01	8.36E-01	
				3	7.11	**2.94E-06**	6.65E-01	
				4	4.95	**2.61E-02**	8.95E-01	
Pink Salmon	2009	N/A	Lower Fraser River	1	13.89	**3.82E-05**	6.67E-01	
				2	9.04	**1.31E-02**	1.44E-01	
				3	5.86	1.44E-01	4.22E-01	
				4	5.48	**2.02E-05**	6.01E-01	

Significant relationships at *P *<* *0.05 are in bold.

The temperature treatments were most significantly associated with PC4 in 2007 and 2009, and PC3 in 2008 (Figure S1B) explaining 5.5–7.1% of the variance in the data (Table 2). Functional groups involved in protein biosynthesis, metabolic processes, cell cycle, antioxidant activity, ATPase activity, immune response, apoptosis, cell proliferation and cell structure were enriched in the PC4 gene lists in 2007 and 2009 (Table S3). Relatively few functional gene groups were enriched in the PC3 gene list in 2008, potentially due to the fact that these fish were not as mature as the fish studied in 2007 and 2009, the overall signal was weaker or there simply were not as many functional groups that varied significantly. Only functional groups involved in RNA processing were enriched in the PC3 gene list in 2008. Sex and population were only significantly associated with PC2 in 2007. Therefore, we suggest that the patterns detected in PC1 (mortality responses) in each year, and PC3 and PC4 (temperature responses) in each year were not sex-or population specific.

### Effects of temperature and sex on the transcriptome

**Figure 3:**
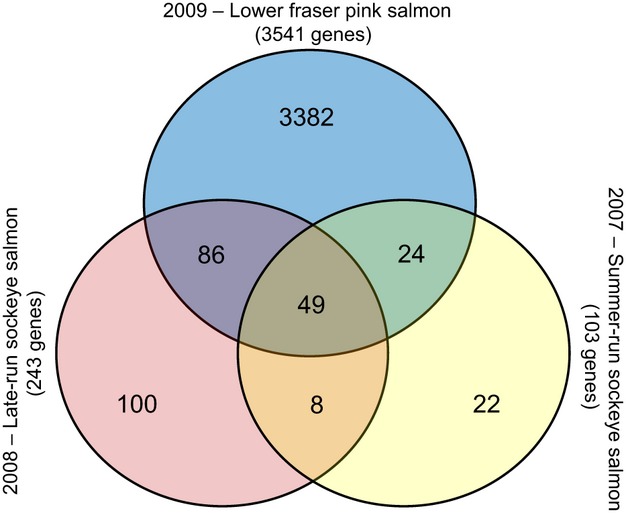
Venn diagram for the Pacific salmon genes that differed significantly in expression levels between warm and cool treatments as determined by the two-factor anovas performed for each experimental year. Numbers represent the number of differentially expressed genes at q < 0.01.

**Figure 4:**
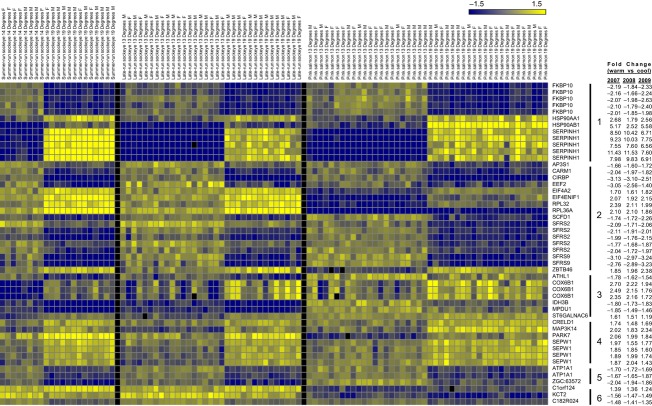
Heat map showing the 49 differentially regulated microarray features common between population and species, and the relative fold change (warm treatment relative to the cool treatment). Genes are grouped based on function (1: Molecular chaperones; 2: Transcription/translation/protein transport; 3: Metabolic processes; 4: Oxidative stress/ion binding/signal transduction; 5: Transmembrane transport; 6: DNA repair, cell structure, no gene symbol available). Gene expression levels in the heat map are presented as normalized log_2_ ratios between an individual fish and the pooled reference. Relative expression levels are indicated by the colour scale, with yellow indicating upregulation and blue indicating down-regulation. Gene symbols or annotation ID's (if gene symbols are not available) are presented along the right side of the heat map.

The effects of temperature and sex were directly compared for each year using two-factor anovas. At *q* < 0.01, 103 microarray features in 2007, 243 features in 2008, and 3541 features in 2009 had significantly different levels of expression between the temperature treatments (Fig. 3). For the gene lists that differed significantly with temperature, 49 features had common expression patterns (i.e. directional change) between both species, and among populations within species. This represents 47.6%, 20.2% and 1.4% of the significant features in 2007, 2008 and 2009 respectively. Of the 49 features, there were 29 unique annotated genes and 1 gene with an unknown function (Fig. 4). Several of the 29 annotated genes [HSP90AB1, HSP90AA1, SERPINH1 (also known as HSP47), EEF2 and CIRBP] are known to be thermally responsive or have been previously determined to be thermally responsive in sockeye salmon using cDNA microarray techniques (e.g. Jeffries et al. 2012a). Of the 49 features, SERPINH1 had the greatest fold change increase in fish exposed to 19°C, followed by HSP90AB1 and HSP90AA1, while FKBP10 and CIRBP had the greatest fold change decreases (Fig. 4). Other significant genes with multiple copies were ATP1A1, COX6B1, SEPW1 and two splicing factors (SFRS2 and SFRS9). In addition to being thermally responsive, the 30 unique genes are generally involved in cell redox homeostasis, protein folding, calcium and ion homeostasis, protein biosynthesis and metabolism. Additional information for the 49 features can be found in Table S4. Within the 19°C treatments, MAP3K14 was consistently positively correlated with post-sampling survival (*P* < 0.05; *r* = 0.60, 0.74, 0.45 in 2007, 2008 and 2009 respectively). Additionally, HSP90AB1 (*r* = 0.64), SFRS2 (two copies; *r* = 0.66 and 0.57) and C1orf124 (*r* = 0.76) were positive correlated and PARK7 (*r* = −0.66) was negatively correlated with post-sampling survival in 2007, COX6B1 (*r* = 0.58) was positively correlated and EIF4A2 (*r* = −0.59) was negatively correlated with post-sampling survival in 2008, and EEF2 (*r* = −0.66) and ST6GALNAC6 (*r* = −0.63) were negatively correlated with post-sampling survival in 2009.

There were no strong differences in gene expression patterns between sexes (only 1, 0 and 14 significantly different features in 2007, 2008 and 2009 respectively). Twelve of the 14 significantly different features in 2009 were transcripts that coded for haemoglobin A and B, which were up-regulated in male pink salmon. The other two features were the 40S ribosomal protein (RPS5; up-regulated in males) and an uncharacterized protein C1orf106 homologue (no gene symbol available; down-regulated in males). In 2007, the only significantly different feature was caveolin-2 (CAV2), which was up-regulated in females. It should be noted that there were unbalanced sex ratios for these analyses in 2007 and 2008 because of the higher overall mortality in female sockeye salmon each year.

## Discussion

For the first time, we present the transcriptomic responses of multiple populations and species of Pacific salmon exposed to high water temperatures for ecologically relevant periods of time in controlled laboratory conditions. Exposure to a 19°C water treatment resulted in higher mortality and the induction of a CSR in both pink and sockeye salmon, and in the different populations of sockeye salmon. Some of the genes involved in the temperature-induced CSR are ‘classic’ heat shock response genes (e.g. HSP90AA1, HSP90AB1, SERPINH1), or are associated with other environmental stressors along with a temperature response (e.g. CIRBP). There are others that have never been previously demonstrated to be associated with a thermal CSR in Pacific salmon (e.g. ATP1A1, FKBP10, SEPW1). Functional analysis showed that genes involved in cell homeostasis, cell cycle regulation and immune response had greater expression with exposure to the high temperature treatment. Previous work has shown that sockeye salmon exposed to high water temperatures have increased expression of genes involved in cell maintenance and immune function (Jeffries et al. 2012a) and this study suggests that this may be a general response to high water temperature for sockeye and pink salmon. Overall, there was greater observed mortality in the 19°C treatment than in the ‘cool’ treatment, which may indicate a link between a temperature-induced CSR and reduced survival in Pacific salmon. Because this CSR was common across these species and populations, these results may also be useful for detecting temperature stress in other populations and species of Pacific salmon.

### Common temperature responses

There was evidence of a common temperature-induced CSR across the pink and sockeye salmon populations studied. With the use of the largest salmonid microarray available (44K genes), along with sample sizes that are excellent for wild salmon genomics research (*n* = 21, 33, and 44 in 2007, 2008 and 2009 respectively), 29 unique annotated genes (and one gene with unknown function) were classified as thermally responsive. Many of the genes that were up-regulated at 19°C were molecular chaperones and were consistent with an unfolded protein response associated with temperature stress. Several heat shock proteins [HSP90AA1, HSP90AB1, SERPINH1] were up-regulated in fish held at 19°C, consistent with patterns previously described in Pacific salmon (Miller et al. 2009; Evans et al. 2011; Jeffries et al. 2012a). Some heat shock proteins are up-regulated during acute exposure to high temperatures; however, during acclimation to chronic high temperature, expression may return to a basal level or be down-regulated (Meyer et al. 2011). This may be species-dependent as HSP90 remained elevated during chronic exposure to extreme water temperatures for the eurythermal annual killifish (Podrabsky and Somero 2004). We found that after an ecologically relevant 5–7 day high water temperature exposure, the heat shock protein response was still activated, which suggests that either these fish could not acclimate to 19°C or that 5–7 days was not a sufficient amount of time for the heat shock proteins to return to basal levels. However, given that adult Pacific salmon have a reduced ability to acclimate to high water temperatures (Clark et al. 2011), and there was increased mortality at 19°C, these gene expression results suggest that the fish were not acclimating to this temperature after 5–7 days of exposure.

There was differential expression of the molecular chaperone FKBP10, down-regulated at 19°C, which has not previously been described as thermally responsive in Pacific salmon. The directional change of FKBP10 was consistent with those of the longjaw mudsucker exposed to a high temperature treatment for 4 weeks (Logan and Somero 2010) and in Atlantic cod (*Gadus morhua*) exposed to a 3-h heat shock treatment (Hori et al. 2010). The consistency between these species may suggest that FKBP10, specific to the endoplasmic reticulum (ER) and involved in calcium ion binding processes, is indicative of a thermal stress response across fish species. Additionally, SERPINH1, an ER resident molecular chaperone involved in collagen stabilization (Krone et al. 1997), was also significantly up-regulated at 19°C. The differential regulation of ER-specific molecular chaperones suggests an activation of the unfolded protein response (reviewed in Rutkowski and Kaufman 2004).

Several genes associated with protein biosynthesis were differentially regulated in response to the temperature treatments. These included the up-regulation at 19°C of an initiation factor (EIF4A2), an initiation factor transporter protein (EIF4ENIF1), 60S ribosomal proteins (RPL32, RPL36A), and a gene involved in transcription regulation (ZBTB46). Interestingly, the upregulation of the EIF4E transporter gene, EIF4ENIF1, detected in this study, may influence protein biosynthesis as EIF4E regulates a rate-limiting step in protein biosynthesis (Sukarieh et al. 2009). Conversely, at 19°C there was a down-regulation of EEF2, (involved in polypeptide elongation), CIRBP (involved in RNA stabilization and translation efficiency), and CARM1 (involved in transcription regulation), along with the splicing factors SFRS2 and SFRS9. The down-regulation of EEF2 and CIRBP during exposure to warm water temperatures is consistent with previous work on sockeye salmon (Jeffries et al. 2012a); however, this study demonstrates that this pattern is common across at least two species of Pacific salmon. Because EEF2 is an essential factor in protein synthesis, its down-regulation may result in an overall reduction in protein biosynthesis. Decreases in protein biosynthesis have been shown in salmonids exposed to a chronic elevated water temperatures and this response may be more pronounced in fish with limited energy availability (Morgan et al. 1999), as would be the case with adult Pacific salmon that rely solely on endogenous energy stores to fuel freshwater migration. The complex variability in the regulation of genes involved in protein biosynthesis suggests that there is an up-regulation of genes required to respond to the temperature stress event (e.g. heat shock proteins), while still resulting in an overall down-regulation of the synthesis of non-essential genes, a common response to periods of ER stress and cellular stress in general (Rutkowski and Kaufman 2004; Storey and Storey 2004). This could be reflected by the fact that genes associated with ER/Golgi-mediated transport mechanisms (AP3S1 and SCFD1), involved in transporting proteins out of the cell, were down-regulated at 19°C.

Oxidative stress is common in organisms during periods of extreme environmental challenges, which includes temperature stress (Kassahn et al. 2009). The hydrogen peroxide sensor gene, PARK7, which protects the cell against oxidative stress (Eltoweissy et al. 2011), along with SEPW1, were significantly up-regulated at 19°C. While SEPW1 is often associated with selenium binding in the cell, it also functions as a glutathione-dependant antioxidant (Whanger 2009). Collectively, the up-regulation of these genes suggests an activation of an oxidative stress response. Indeed, functional categories representing an oxidative stress response were significantly enriched in the 2009 gene list. Ageing, senescence and disease also contribute to oxidative stress and susceptibility to oxidative stress (Ermak and Davies 2002; Martinez-Alvarez et al. 2005); these factors were likely stronger contributors in the 2007 and 2009 studies as these fish were closer to final maturation than the late-run sockeye salmon sampled in 2008.

Oxidative stress can lead to fluctuations in cellular calcium levels that can cause physiological disturbances (protein phosphorylation) and apoptosis (Ermak and Davies 2002). Indeed, MAP3K14, which specifically regulates the stress responsive nuclear factor kappa-B (NFkB) pathway through protein phosphorylation, was up-regulated at 19°C. Interestingly, MAP3K14 has been linked to inhibiting apoptosis in cells during an oxidative stress response (Wixted et al. 2010) and was positively correlated with survival in Pacific salmon held at 19°C in the present study. Therefore, MAP3K14 levels may be predictive of salmon survival during periods of high water temperatures. Additionally, genes associated with cellular calcium binding and homeostasis (FKBP10, CRELD1) were differentially affected by the temperature treatments suggesting that cellular calcium-mediated processes may be important for the temperature response in Pacific salmon. However, given the diverse role of calcium in cell function, it is difficult to make any direct associations between calcium signalling and the oxidative stress response in the present study; therefore, further investigation into the role of calcium signalling in the Pacific salmon CSR is required.

The Na^+^/K^+^-ATPase protein, ATP1A1, was down-regulated in Pacific salmon held at 19°C. Sockeye salmon held at high temperatures for 24 days have been shown to have decreased total Na^+^/K^+^-ATPase activity (Crossin et al. 2008). Because Na^+^/K^+^-ATPase can use a significant portion of the cellular energy budget, a down-regulation of this process may be used to conserve energy at the cellular level in organisms (Staples and Buck 2009). This response to environmental stress may be particularly strong in organisms with limited endogenous energy stores (Richards 2010), such as migrating Pacific salmon. There would be a need to maintain proper electrochemical gradients across membranes following a down-regulation of ATP1A1 otherwise the end result of this potentially energy conserving mechanism could be a disturbance of ion homeostasis. We have shown previously that plasma chloride and osmolality levels were elevated in the high temperature treatment, suggesting an osmoregulatory disturbance, in a subset of sockeye salmon from these same holding experiments (Jeffries et al. 2012a,b2012b) that may be associated with a down-regulation of ATP1A1 as detected in the present study.

The temperature-induced CSR appears to be an adaptive response to exposure to high water temperature stress as it is associated with improved short-term survival. Individuals from the warm water treatment that clustered with individuals from the cool water treatment in the PCA died shortly after sampling. This suggests that those fish did not have an appropriate response to the high water temperature which could be due to inter-individual differences in thermal tolerance, influences of disease or parasites, or because these fish were further along the senescence trajectory. Variable responses to high water temperature associated with elevated mortality have been previously observed in sockeye salmon gene expression profiles (Miller et al. 2009; Jeffries et al. 2012a) and suggest that a lack of a CSR by an individual is predictive of temperature-induced mortality in Pacific salmon.

### Sex differences

We did not detect strong effects of sex on the transcriptomes, which is surprising given recent evidence of sex-specific mortality patterns in Pacific salmon (e.g. Crossin et al. 2008; Keefer et al. 2010; Jeffries et al. 2012b) and in this study. However, the causes of these sex-specific differences in mortality do not appear to be detected at the level of the transcriptome, a finding that is supported by previous transcriptomics work conducted on adult sockeye salmon (Miller et al. 2009; Jeffries et al. 2012a). It should be noted that these gill samples were taken from non-perfused gills and therefore patterns detected could be due to transcription in the gill as well as in blood in the gill tissue (Quinn et al. 2011b), which could lead to the detection of haemoglobin genes. This may explain the differences in the expression of haemoglobin A and B between male and female pink salmon detected in the present study. These differences in haemoglobin transcript levels support previous observations of increased blood haemoglobin concentration in maturing male pink salmon (T.D. Clark, personal communication), a trend that is reversed in sockeye salmon (Clark et al. 2009). It is possible that the up-regulation of haemoglobin genes in male pink salmon may be required for aerobic processes associated with aggressive encounters with other males during maturation and spawning.

### Species differences

There were differences in the relative effect of the high temperature treatment on the transcriptomes of the different species in this study. There were >14 times more genes differentially regulated in response to high temperature exposure (3541 features, with 3382 features unique to pink salmon) in pink salmon compared with sockeye salmon, which may be partially reflective of the larger sample sizes in the 2009 experiment. It is possible that there were species-specific differences in hybridization to the array as to our knowledge, these are among the first data that hybridized samples from pink and sockeye salmon to these particular arrays. However, the fact that there were consistent changes in gene expression due to the temperature treatments between pink and sockeye salmon, it is unlikely that discrepancies in hybridization to the array account for all of the differences in the transcriptomic responses between species. Pink salmon had up-regulated genes involved in oxidative stress and calcium-mediated processes, along with lipid metabolism and protein catabolism that were not detected in sockeye salmon. There could be several causes of this pattern. Recent work suggests that pink salmon have a higher aerobic capacity, enabling them to be more resilient to warm temperatures (Clark et al. 2011). However, they demonstrate an exaggerated thermal and handling stress response (based on blood plasma indices) compared with sockeye salmon (Jeffries et al. 2012b; M.R. Donaldson, Ph.D. Thesis, University of British Columbia). It should be noted though, that the fish used in the Clark et al. (2011) study were less reproductively mature than the fish used in the present study and were subjected to fluctuating acute temperatures rather than chronic temperature exposures. Consistent with higher blood plasma stress indices observed in a subset of fish from the same holding study (e.g. glucose and lactate; Jeffries et al. 2012b), these gene expression patterns could indicate that the temperature stressor, in combination with handling and confinement stresses, resulted in a relatively more stressful holding period for the pink salmon. This increased stress response may have led to the differential expression of genes involved in an oxidative stress response and calcium-mediated processes. In addition, chronic exposure to environmental stress may result in alterations in the expression of genes involved in fatty acid metabolism (McClelland 2004) a pathway that was only disrupted in pink salmon. Collectively, we suggest that the pink salmon were more stressed overall in this study.

We were only able to sample one species and population each year due to logistical constraints, therefore any conclusions regarding a species effect must be viewed cautiously. Because there can be substantial inter-annual variation within populations, it is currently unknown whether the responses detected in the present study are consistent among years for those populations. Future work should therefore examine the temperature-induced CSR across multiple cohorts of the same population. Additionally, the differences detected may have been influenced by the life-history characteristics (i.e. spawning dates) of the populations considered. To adequately test for species level differences, multiple populations from both species would need to be compared during the same holding experiment. Logistically, this would be very difficult to accomplish; however, future research should move in this direction to be able to fully determine if species and population level differences in Pacific salmon temperature-induced CSRs exist.

### Effects of senescence and maturation

Processes associated with senescence and mortality appeared to be detectable in gene expression profiles of the adult Pacific salmon in this study. Survival post-sampling was correlated with PC1 in each year and with PC2 in the PCA across years and species. Functional analysis of PC1 in each year shows that genes involved in cell proliferation and apoptosis were enriched in the PC1 gene lists; these processes are ultimately associated with cell death (Rutkowski and Kaufman 2004). Genes involved in an immune response and ion homeostasis were also associated with PC1. Immunosuppression leading to increased susceptibility to disease is known to occur in senescing and moribund salmon (Dickhoff 1989; Miller et al. 2009; Jeffries et al. 2012a), with disease likely being the ultimate cause of death (Gilhousen 1990). The freshwater component of spawning migrations is also characterized by a gradual loss of osmoregulatory ability (Shrimpton et al. 2005), followed by a sharp decrease in plasma ion levels days in advance of actual death (Jeffries et al. 2011), and in this study, differential regulation of genes associated with ion homeostasis. There is potential that these osmoregulatory shifts, which occur in advance of death, may be predictive of mortality in Pacific Salmon.

The mortality and gene expression patterns could have been influenced by the proximity to final maturation and processes associated with natural senescence. Functional analysis of the PC most associated with the temperature response for each year suggested that there are additional effects of temperature stress on fish that are closer to final maturation and further along the senescence trajectory. Proximity to final maturation has been shown to influence the effect of temperature on survival in a subset of these pink and sockeye salmon (Jeffries et al. 2012b). At the time of the temperature holding experiments, the summer-run sockeye salmon in 2007 and pink salmon in 2009 were closer to final maturation than the late-run sockeye salmon from the 2008 experiment. Indeed, some of the differences in relative expression of certain genes between the temperature treatments were greater in the fish used in the 2007 and 2009 studies compared with the 2008 study (e.g. FKBP10, HSP90AA1, HSP90AB1). Proximity to final maturation may have resulted in differential regulation of genes involved in maintenance of the cell cycle and apoptosis, cellular and oxidative stress response, and various metabolic processes. These functional groups were not enriched in 2008, fish that were over a month away from the peak spawning period for that population. The response of genes associated with apoptosis and oxidative stress may be related to processes involved in natural senescence (Miller et al. 2009; Jeffries et al. 2012a) or an interaction between senescence and the temperature stress response.

## Conclusion

High premature mortality was coupled with a CSR signature in different populations and species of Pacific salmon. Despite knowledge that various species and populations of Pacific salmon have different aerobic capabilities at elevated water temperatures (Clark et al. 2011; Eliason et al. 2011), which has been used as a proxy for differences in thermal tolerance, a threshold temperature of 18°C (Macdonald et al. 2000) is often used for determining when spawning migrations are considered more difficult due to temperature stress for all populations of Fraser River Pacific salmon. Indeed, migration during temperatures >18°C results in increased *en route* mortality for some populations of sockeye salmon (Martins et al. 2011). This threshold temperature has often been reached or exceeded in recent years and this study demonstrates that a common CSR is induced when temperatures exceed 18°C for sockeye and pink salmon. These data suggest that despite the different evolutionary histories of these species, components of the temperature-induced CSR are conserved, and these results may be a useful step towards developing biomarkers of temperature stress that can be applied to other species and populations of Pacific salmon.

It is currently unknown whether chronic activation of a CSR is harmful for Pacific salmon. However, given that chronic exposure to 18–19°C water temperature results in increased mortality in sockeye and pink salmon (Crossin et al. 2008; Jeffries et al. 2012b; this study) and can result in osmoregulatory disturbances in sockeye salmon (Jeffries et al. 2012a,b2012b), it appears that Pacific salmon that experience chronic temperatures capable of inducing a CSR and other physiological responses are at an elevated risk of dying prematurely. The ultimate cause of death is still unknown, but sublethal high water temperatures interact with disease and parasite progression, which are often considered factors in the actual death of senescing Pacific salmon (Gilhousen 1990). In conclusion, a frequently encountered water temperature of 19°C will induce a CSR in pink and sockeye salmon, which may increase the risk of premature mortality for individuals of these species.

## Data archiving statement

The microarray data have been deposited in the Gene Expression Omnibus (http://www.ncbi.nlm.nih.gov/geo/) with the accession number GSE42558.

## Supporting Information

Figure S1 Heat maps showing the top 20 positively and top 20 negatively loaded genes for the principal component (PC) most related with (A) survival (PC1 for each year; mortality date is indicated) and (B) temperature (PC4 for 2007 and 2009; PC3 for 2008; temperature is indicated).

**Table S1**. Functional analysis performed using the receiver operator characteristic (ROC) scoring method in ErmineJ for the first three principal components (PC) of the principal component analysis performed on all experimental years. **Table S2**. Functional analysis performed using the receiver operator characteristic (ROC) scoring method in ErmineJ for the principal component (PC) most associated with survival (PC1 for each year) for the principal component analysis performed on each individual experimental year. **Table S3**. Functional analysis performed using the receiver operator characteristic (ROC) scoring method in ErmineJ for the principal component (PC) most associated with temperature (PC4 for 2007 and 2009, PC3 for 2008) for the principal component analysis performed on each individual experimental year. **Table S4**. Annotation and gene function information for the 49 differentially expressed microarray features common between population and species.
