# Supplementary material for: Transcriptomic responses to high water temperature in two species of Pacific salmon
Source: Evol Appl. 2013 Nov 12;7(2):286–300. doi: 10.1111/eva.12119 (PMC3927889; doi:10.1111/eva.12119)
Supplement: Figure S1 — Heat maps showing the top 20 positively and top 20 negatively loaded genes for the principal component (PC) most related with (A) survival (PC1 for each year; mortality date is indicated) and (B) temperature (PC4 for 2007 and 2009; PC3 for 2008; temperature is indicated). [file eva0007-0286-sd1.pdf]

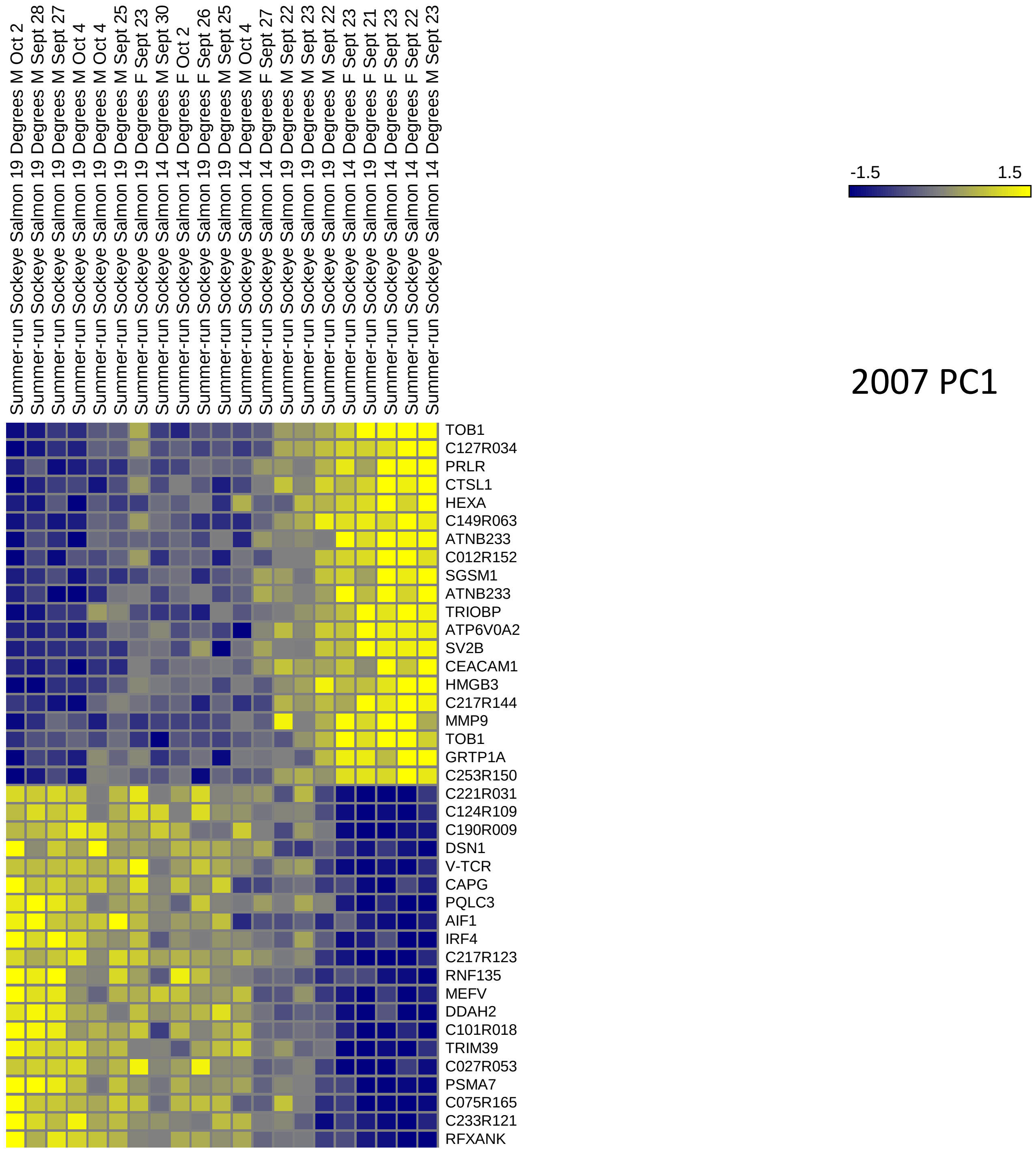

2007 PC1

Figure S1A

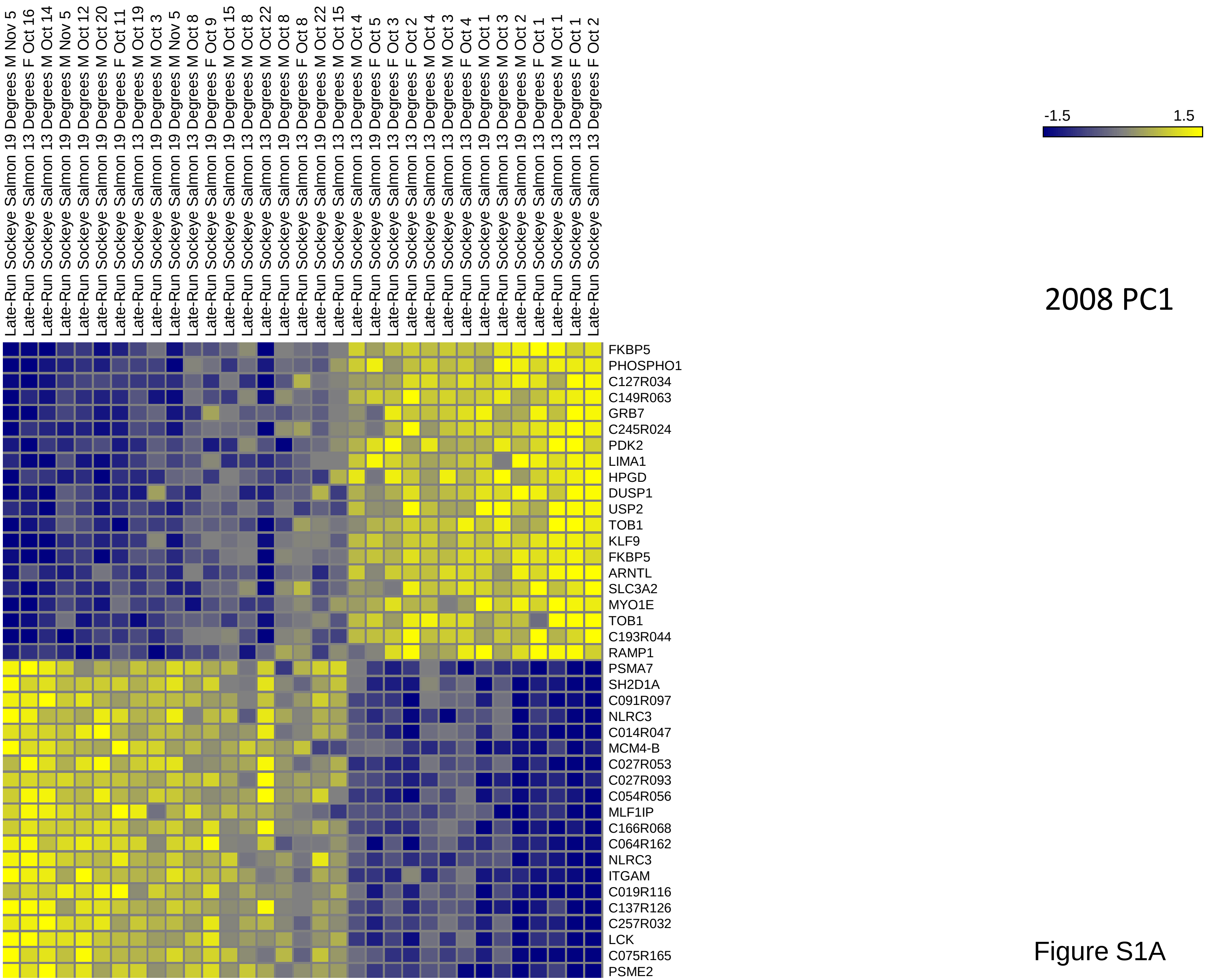
Late-Run Sockeye Salmon 19 Degrees M Nov 5
Late-Run Sockeye Salmon 13 Degrees F Oct 16
Late-Run Sockeye Salmon 13 Degrees M Oct 14
Late-Run Sockeye Salmon 19 Degrees M Nov 5
Late-Run Sockeye Salmon 19 Degrees M Oct 12
Late-Run Sockeye Salmon 19 Degrees M Oct 20
Late-Run Sockeye Salmon 19 Degrees F Oct 11
Late-Run Sockeye Salmon 13 Degrees M Oct 19
Late-Run Sockeye Salmon 19 Degrees M Oct 3
Late-Run Sockeye Salmon 19 Degrees M Nov 5
Late-Run Sockeye Salmon 13 Degrees M Oct 8
Late-Run Sockeye Salmon 19 Degrees F Oct 9
Late-Run Sockeye Salmon 19 Degrees M Oct 15
Late-Run Sockeye Salmon 13 Degrees M Oct 8
Late-Run Sockeye Salmon 13 Degrees M Oct 22
Late-Run Sockeye Salmon 19 Degrees M Oct 8
Late-Run Sockeye Salmon 13 Degrees F Oct 8
Late-Run Sockeye Salmon 19 Degrees M Oct 22
Late-Run Sockeye Salmon 13 Degrees M Oct 15
Late-Run Sockeye Salmon 13 Degrees M Oct 4
Late-Run Sockeye Salmon 19 Degrees F Oct 5
Late-Run Sockeye Salmon 13 Degrees F Oct 3
Late-Run Sockeye Salmon 13 Degrees F Oct 2
Late-Run Sockeye Salmon 13 Degrees M Oct 4
Late-Run Sockeye Salmon 13 Degrees M Oct 3
Late-Run Sockeye Salmon 13 Degrees F Oct 4
Late-Run Sockeye Salmon 19 Degrees M Oct 1
Late-Run Sockeye Salmon 13 Degrees M Oct 3
Late-Run Sockeye Salmon 19 Degrees M Oct 2
Late-Run Sockeye Salmon 13 Degrees F Oct 1
Late-Run Sockeye Salmon 13 Degrees M Oct 1
Late-Run Sockeye Salmon 13 Degrees F Oct 1
Late-Run Sockeye Salmon 13 Degrees F Oct 2
FKBP5
PHOSPHO1
C127R034
C149R063
GRB7
C245R024
PDK2
LIMA1
HPGD
DUSP1
USP2
TOB1
KLF9
FKBP5
ARNTL
SLC3A2
MYO1E
TOB1
C193R044
RAMP1
PSMA7
SH2D1A
C091R097
NLRC3
C014R047
MCM4-B
C027R053
C027R093
C054R056
MLF1IP
C166R068
C064R162
NLRC3
ITGAM
C019R116
C137R126
C257R032
LCK
C075R165
PSME2
-1.5
1.5
2008 PC1

Figure S1A

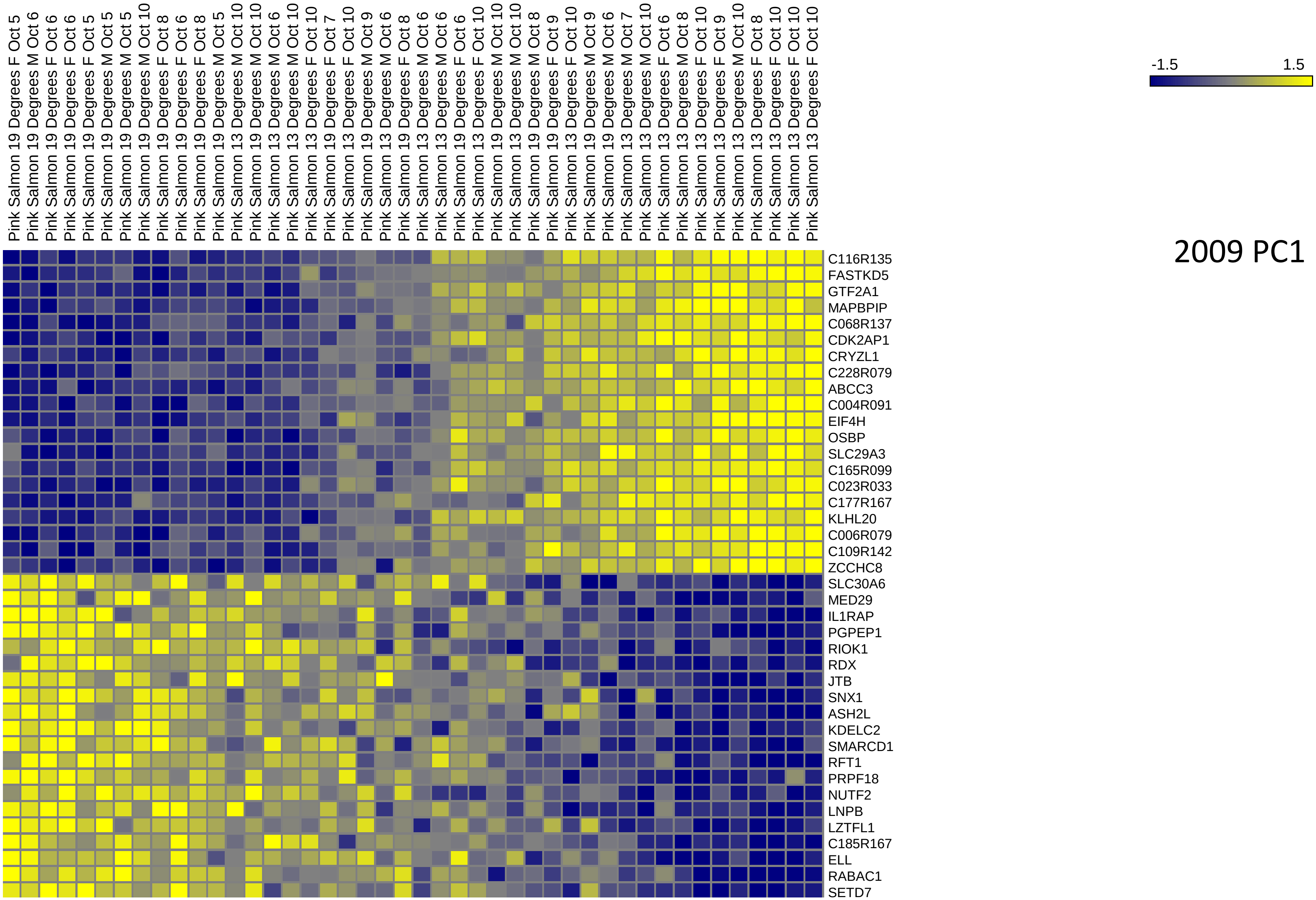
Pink Salmon 19 Degrees F Oct 5
Pink Salmon 19 Degrees M Oct 6
Pink Salmon 19 Degrees F Oct 6
Pink Salmon 19 Degrees F Oct 6
Pink Salmon 19 Degrees F Oct 5
Pink Salmon 19 Degrees M Oct 5
Pink Salmon 19 Degrees M Oct 5
Pink Salmon 19 Degrees M Oct 10
Pink Salmon 19 Degrees F Oct 8
Pink Salmon 19 Degrees F Oct 6
Pink Salmon 19 Degrees F Oct 8
Pink Salmon 19 Degrees M Oct 5
Pink Salmon 13 Degrees M Oct 10
Pink Salmon 19 Degrees M Oct 10
Pink Salmon 13 Degrees M Oct 6
Pink Salmon 13 Degrees M Oct 10
Pink Salmon 13 Degrees F Oct 10
Pink Salmon 19 Degrees F Oct 7
Pink Salmon 13 Degrees F Oct 10
Pink Salmon 19 Degrees M Oct 9
Pink Salmon 13 Degrees M Oct 6
Pink Salmon 19 Degrees F Oct 8
Pink Salmon 13 Degrees M Oct 6
Pink Salmon 13 Degrees M Oct 6
Pink Salmon 19 Degrees F Oct 6
Pink Salmon 13 Degrees F Oct 10
Pink Salmon 19 Degrees M Oct 10
Pink Salmon 13 Degrees M Oct 10
Pink Salmon 19 Degrees M Oct 8
Pink Salmon 19 Degrees F Oct 9
Pink Salmon 13 Degrees F Oct 10
Pink Salmon 19 Degrees M Oct 9
Pink Salmon 19 Degrees M Oct 6
Pink Salmon 13 Degrees M Oct 7
Pink Salmon 13 Degrees M Oct 10
Pink Salmon 13 Degrees F Oct 6
Pink Salmon 13 Degrees M Oct 8
Pink Salmon 13 Degrees F Oct 10
Pink Salmon 13 Degrees F Oct 9
Pink Salmon 13 Degrees M Oct 10
Pink Salmon 13 Degrees F Oct 8
Pink Salmon 13 Degrees F Oct 10
Pink Salmon 13 Degrees F Oct 10
Pink Salmon 13 Degrees F Oct 10
C116R135
FASTKD5
GTF2A1
MAPBPIP
C068R137
CDK2AP1
CRYZL1
C228R079
ABCC3
C004R091
EIF4H
OSBP
SLC29A3
C165R099
C023R033
C177R167
KLHL20
C006R079
C109R142
ZCCHC8
SLC30A6
MED29
IL1RAP
PGPEP1
RIOK1
RDX
JTB
SNX1
ASH2L
KDELC2
SMARCD1
RFT1
PRPF18
NUTF2
LNPB
LZTFL1
C185R167
ELL
RABAC1
SETD7
-1.5
1.5
2009 PC1

Figure S1A

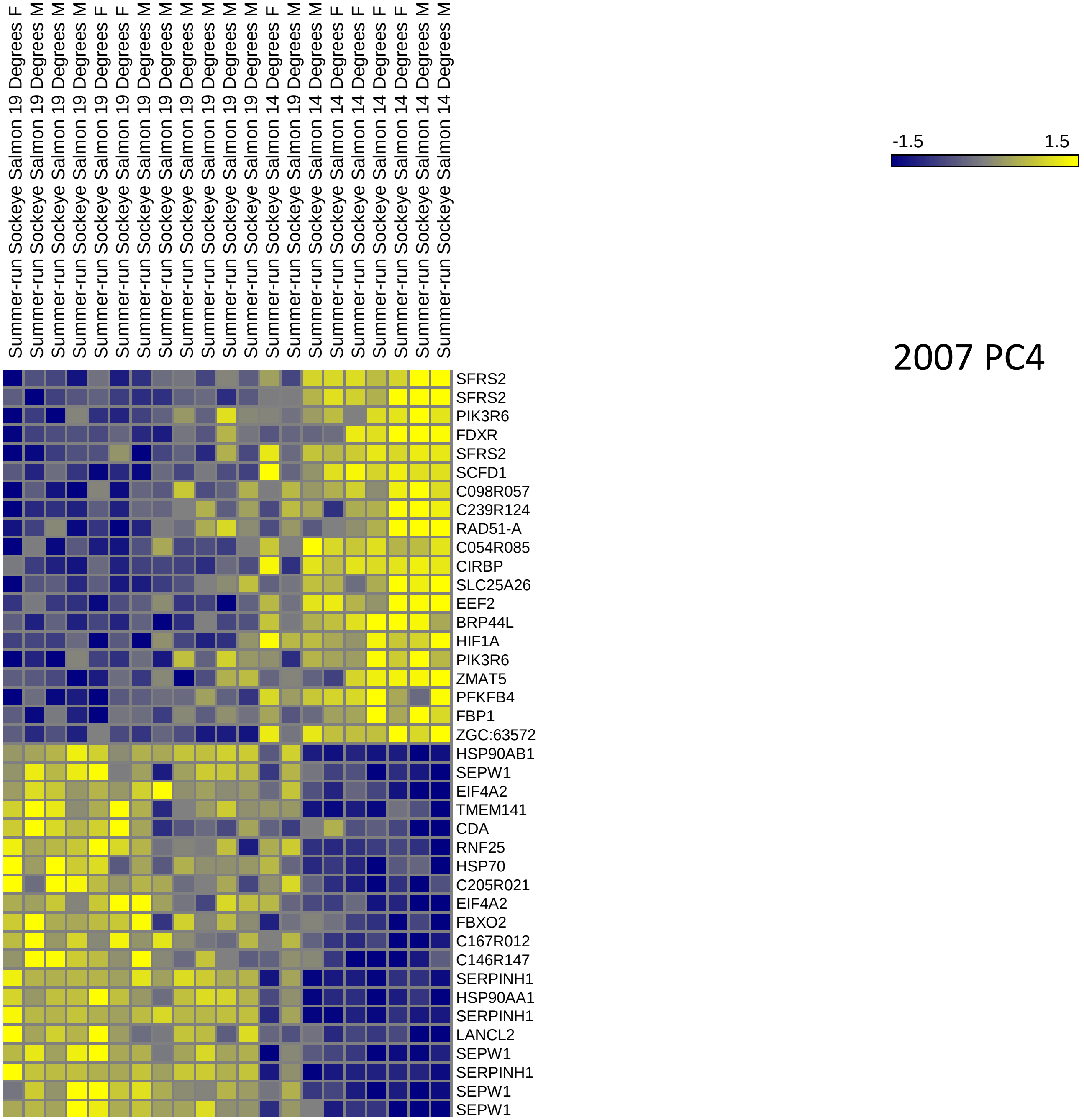

2007 PC4

Figure S1B

2008 PC3

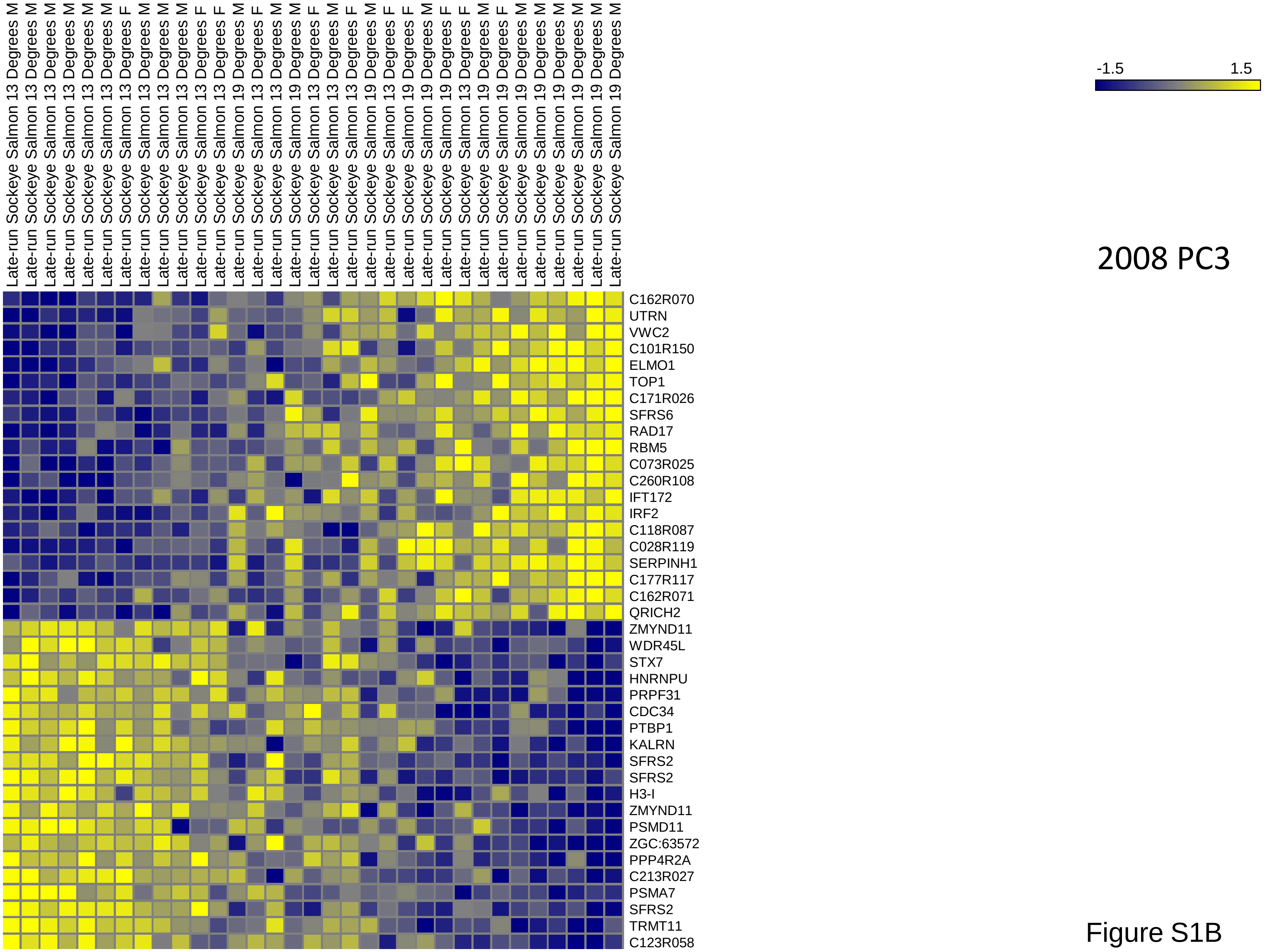

Figure S1B

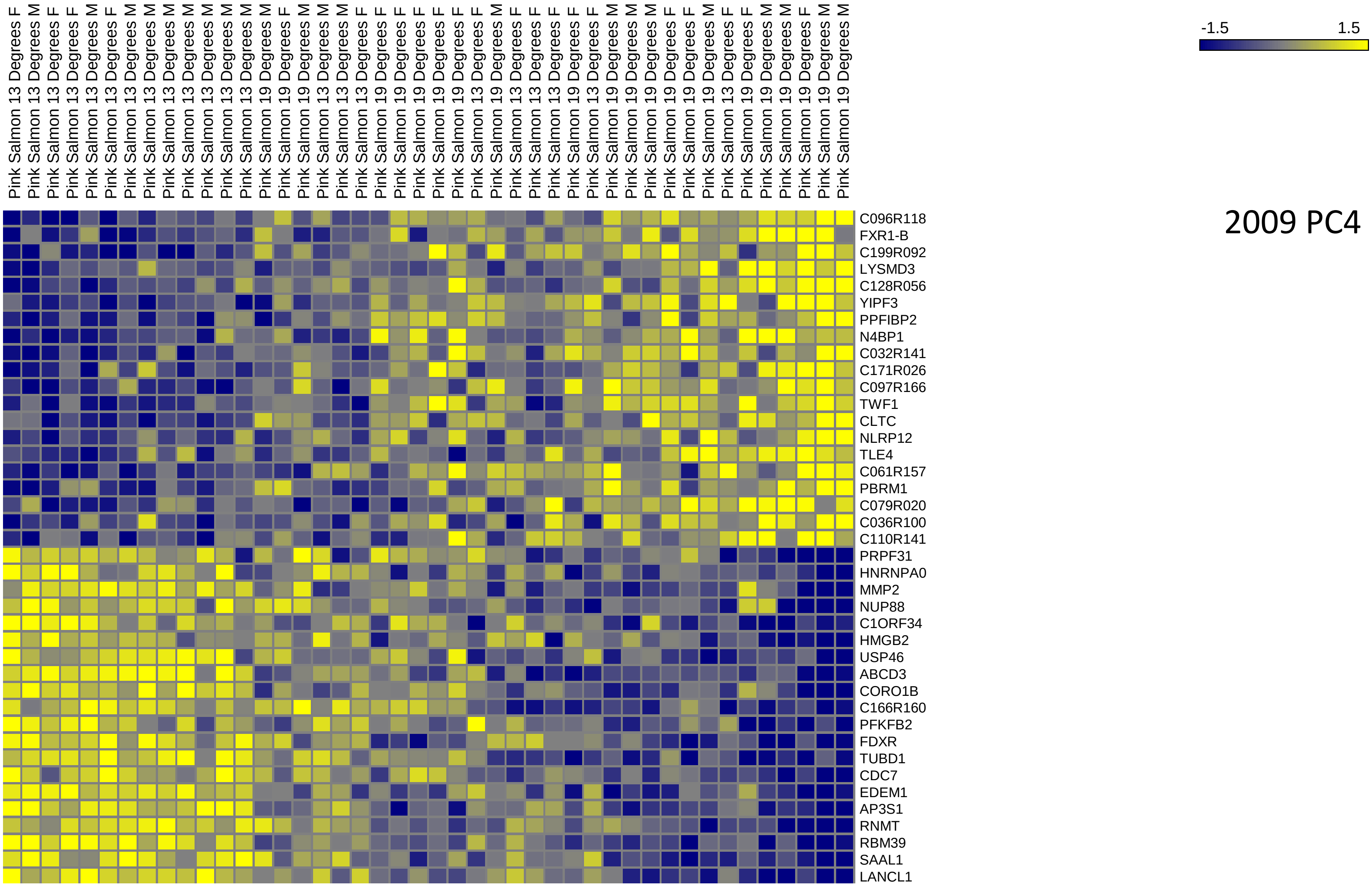

2009 PC4

Figure S1B
